# A novel *NCOR2-NTRK1* fusion detected in a patient of lung adenocarcinoma and response to larotrectinib: a case report

**DOI:** 10.1186/s12890-021-01490-x

**Published:** 2021-04-17

**Authors:** Lei Zhang, Huanhuan Liu, Ye Tian, Huina Wang, Xueying Yang

**Affiliations:** 1 Department of Thoracic Surgery, The Fourth Affiliated Hospital of China Medical University, 4 Chongshan East Road, Shenyang, 110032 Liaoning People’s Republic of China; 2 Acornmed Biotechnology Co., Ltd., 13 Kechuang Road, Beijing, 100176 People’s Republic of China

**Keywords:** Lung adenocarcinoma, NTRK fusion, PD-L1, Larotrectinib, Case report

## Abstract

**Background:**

The identification of NTRK fusions in tumours has become critically important due to the actionable events predictive of response to TRK inhibitor. It is not clear whether the NTRK breakpoint location is different for response to targeted therapy and NTRK fusions affects the efficacy of immunotherapy.

**Case presentation:**

Here we reported a 60-year-old female diagnosed with advanced lung adenocarcinoma. NGS-based molecular profiling identified a novel NCOR2-NTRK1 fusion and high tumor mutational burden (TMB) (58.58 mutations/Mb) in this case. Additionally, program death-ligand 1 (PD-L1) expression was detected in 20–30% of the tumor cells by immunohistochemical (IHC) staining. The patient received treatment with anti-PD-1 immune checkpoint inhibitor of camrelizumab. After two cycles of treatment, the CT scan showed some tumor nodules were still enlarged, indicating disease progression. She was then changed to TRK inhibitor larotrectinib. One month later, the CT scan showed the volume of some lesions started to decrease, and no metastasis lesions were found. The patient then continued the administration of larotrectinib, and some lesion sizes were significantly reduced or even disappeared in the next few months. Currently, this patient is still alive.

**Conclusions:**

Altogether, this report provided a new driver of lung adenocarcinoma expanded the mutational spectrum of NTRK1 fusion variants and suggested using larotrectinib as the targeted therapy is more effective than anti-PD-1 inhibitor in lung adenocarcinoma harboring with NTRK fusion, positive PD-L1 expression, and high TMB simultaneously.

## Background

Lung is a type of molecular-based cancer treatment in personalized medicine. These therapies involve drugs or substances which could interfere with the specific molecules such as fusion genes or mutated genes to exert functions in the cell growth, progression, and spreading of cancer cells, which do not affect the normal cells [1]. Recently, *NTRK* fusions have been identified as new targets of cancer therapy for TRK inhibitors, such as larotrectinib and entrectinib. Immunotherapy, as a research hotspot in recent years, has been approved in many cancer species, including non-small cell lung cancer. However, it is not clear whether the presence of NTRK fusion affects the efficacy of immunotherapy.

In this report, we presented a case of advanced lung adenocarcinoma for whom tumor tissue samples underwent NGS and IHC, and several druggable alterations were identified. Among them, a novel *NCOR2-NTRK1* fusion, positive PD-L1 expression, and high TMB were found. By administration of the TRK inhibitor larotrectinib, the tumor had a partial response after treatment for 5 months and still survives currently.

## Case presentation

**Fig. 1:**
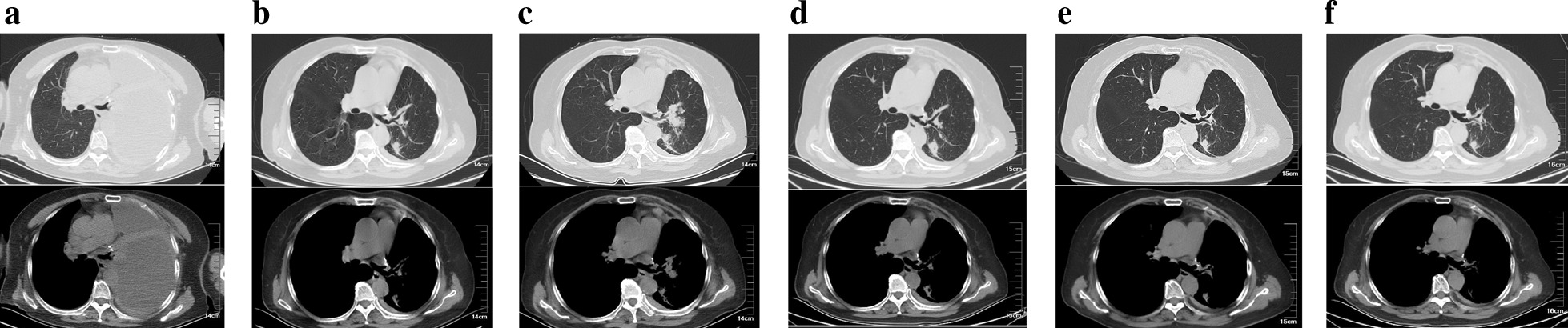
The chest computed tomography (CT) scan images showing the locations of tumor lesions found in **a** left pleural effusion of lung when the patient presented with dyspnea in June 2019; **b** after thoracentesis, two metastatic nodules in the left lower lobe; **c** after accepted PD-1 inhibitor Camrelizumab, 200 mg/time, twice per week, for a total of 4 injections (2 cycles), the tumor nodules were still increased, indicating the disease progressed; **d** After taking larotectinib, 200 mg/day, orally for 1 month, some lesions showed decreased in size; **e** oral administration of larotectinib for 3 months indicated some cancerous lesions were continuously reduced in size; **f** Oral administration of larotectinib for 6 months showed that some cancerous lesions either are reducing or even disappeared

**Fig. 2:**
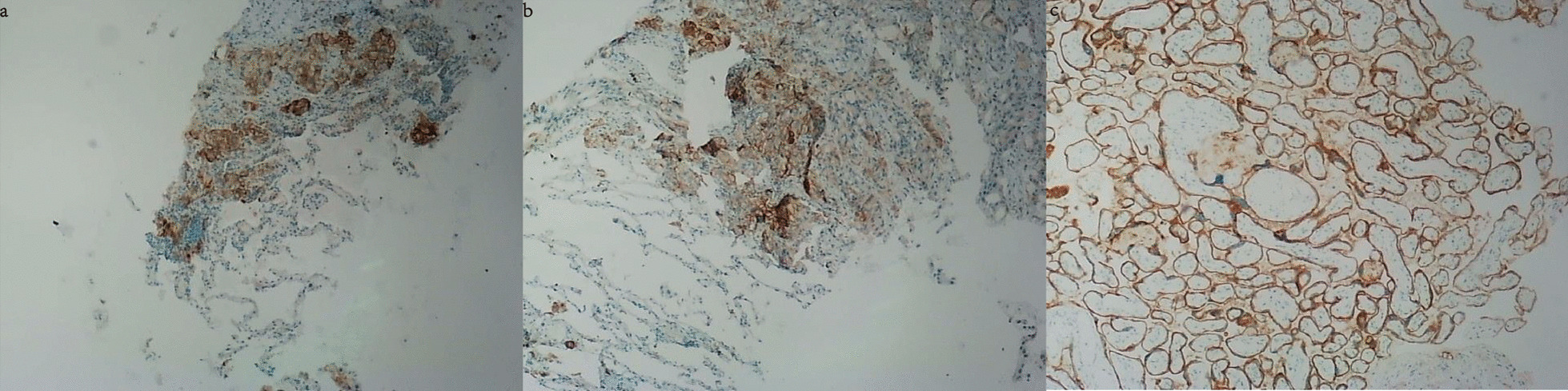
Immunohistochemical (IHC) analysis showed PD-L1 was expressed in 20–30% of lung tumor cells of this patient (**a**, **b**) as compared with the positive control (**c**). The antibody of PD-L1 (VentanaSP263) was purchased from Roche, San Francisco, CA, USA)

**Fig. 3:**
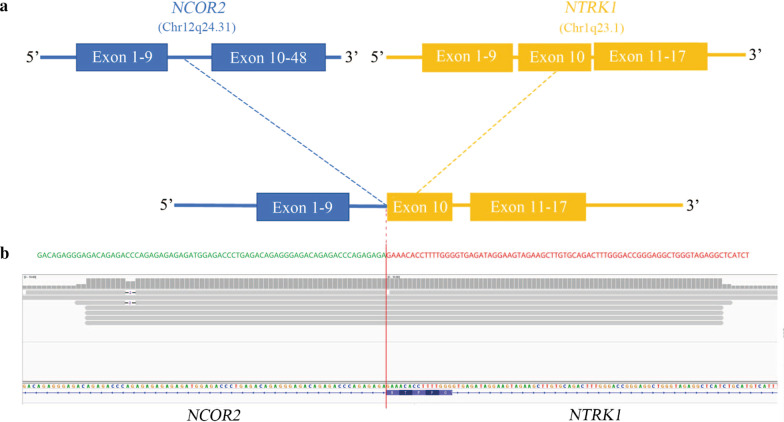
Next-generation sequencing findings for the tumor tissue sample. **a** A novel intergenic region between *NCOR2* and *NTRK1* fusion variant was identified, and **b** next-generation sequencing results showing break point of *NCOR2-NTRK1* fusion

In October 2015, a 60-year-old non-smoker and non-alcoholic woman was admitted to our hospital due to the tumor found at the upper lobe of the left lung, who then underwent left upper lobe resection and left mediastinal hilar lymphadenectomy, and was diagnosed with lung adenocarcinoma with stage IIIA (T2aN2M0). After the operation, the patient refused all adjuvant treatments and did not turn up to the clinical examination on time. In June 2019, the patient showed symptoms of dyspnea. A chest computed tomography (CT) scan showed atelectasis in the left lower lobe of the lung and a massive pleural effusion in the left chest, but no metastatic lesions were found in other organs (Fig. 1a). After the pleural effusion was drawn, a further CT scan found that the metastatic nodules existed in the lower lobe and pleura of the left lung (Fig. 1b). Therefore, the tumor was stage IV. The patient subsequently underwent molecular tests, including NGS and IHC analyses to guide subsequent treatment. The results showed that the PD-L1 expression of the tumor cells was 20–30% (Fig. 2), TMB was 58.58 mutations/Mb, and intriguingly, a novel *NCOR2-NTRK1* fusion was detected (Table 1, Fig. 3). This novel *NTRK1* fusion has not been reported thus far. Since larotrectinib was not available at that time, and the patient refused chemotherapy. This patient started the treatment of PD-1 inhibitor, camrelizumab (Jiangsu Hengrui Medicine Co., Ltd, China) (200 mg/time, twice per week) alone in a clinical trial at other hospitals from July 2019. However, in September 2019, a chest CT scan revealed that the metastatic nodules still enlarged, indicating disease progression (Fig. 1c). We then switched to TRK inhibitor larotrectinib (200 mg/day). After 1 month, the chest CT scan showed that the volume of some lesions was reduced by 30%, indicating larotrectinib was a useful therapeutic option to this patient (Fig. 1d). We then continued to treat the case with larotrectinib. After another 2 months, the CT examination showed that some lesion sizes was reduced continuously by 50%, and no metastatic lesions in other organs were found (Fig. 1e). In March 2020, the further CT scan showed that some lesions were remarkably reduced or even disappeared (Fig. 1f). To December 2020, the patient continues to take larotrectinib and is still alive.

**Table 1:** Genetic alterations identified in the tumor by next-generation sequencing

Gene	Protein variant
*ERBB2*	p.T862A
*NF1*	Splicing
*NTRK1*	*NCOR2-NTRK1* fusion
TMB	58.58 mutations/Mb, high TMB

## Discussion and conclusions

Advances in genomic research have promoted the development of personalized medicine for the treatment of lung cancer [2]. In this study, we studied the genetic profiling and treatment options for a 60-year-old female patient with advanced lung adenocarcinoma. By NGS-based molecular profiling and IHC analyses, we identified several potentially targetable genetic alterations, including *NCOR2-NTRK1* fusion, *ERBB2*, and *NF1*mutations, as well as high levels of TMB and PD-L1 expression, which provided us some therapeutic options for this patient.

Based on the analysis of NGS, tumors could be defined as high and low TMB determined by the prevalence of somatic mutations in their genome [3]. In this case, the genomic profiling revealed the TMB was 58.58 mutations/Mb, and regarded as high TMB (Table 1), suggesting that this case is a genomically unstable tumor type. Previous studies reported that NSCLC patients with high TMB was associated with better survival outcomes of immune checkpoint inhibitors (ICI) therapy [4]. Recent evidence has suggested the use of programmed death-1 (PD-1) and PD-L1 inhibitors remarkably improves response rate of advanced metastatic NSCLC compared to the traditional chemotherapy [5]. On June 2020, the Food and Drug Administration granted accelerated approval to pembrolizumab (KEYTRUDA, Merck & Co., Inc.) for the treatment of adult and pediatric patients with unresectable or metastatic TMB-H solid tumors, which further indicated the important role of TMB in ICI therapy. Additionally, this patient also had 20–30% tumor cells expressing PD-L1. Thus, the patient was treated by camrelizumab (PD-1 inhibitor) which showed high efficacy and good response in breast cancer [6]. However, 2 months after the patient received treatment, the CT showed an enlarged tumor. Although we cannot rule out the possibility that the initial "lack" of response to camrelizumab could have been that effect was not seen yet after 2 months of therapy, we tend to think that tumor suppression effect of camrelizumab was failed. In addtion, based on clinical trial design, the patient was removed from the clinical trial.Gainor et al. reported that patients with *EGFR* mutations and *ALK* fusions had low response rates to PD-1/PD-L1 inhibitors compared than those in EGFR wild-type/ALK-negative patients in Non-Small Cell Lung Cancer (NSCLC) [7]. Hong et al. demonstrated that upregulation of PD-L1 by ALK fusion protein mediates the immune escape by inducing the apoptosis of T cells [8]. Based on the finding, a possible explanation for this unexpected finding could be driver gene of *NCOR2-NTRK1* fusion could be causing resistance to PD-1/PD-L1 blockade immunotherapy, which needs to be further explored.

The TRK family of receptor tyrosine kinases are encoded by *NTRK1*, *NTRK2*, and *NTRK3* genes, and maintain normal functions and development of the nervous system [9]. The *NTRK* fusions in cancers have attracted the attention of many investigators because these *NTRK* fusions-associated cancers are susceptible to NTRK-TKIs [10]. Importantly, these TKIs either tested in phase III clinical trials or are commercially available [10]. *NTRK1* gene rearrangements were firstly found in a small population of lung cancer patients, and this gene is frequently fused with several fusion partners in NSCLC (Table 2) [11]. Compared to *NTRK1*, *NTRK2,* and *NTRK3* rearrangements are relatively rare but are still found in lung cancers (Table 2). In this study, we found that the exon 9 of *NCOR2* fused to the exon 9 of *NTRK1* gene to generate a novel *NTRK1* fusion protein (*NCOR2-NTRK1*). *NCOR2* (Nuclear Receptor Corepressor 2) gene functions as a co-repressor for mediating transcriptional silencing of specific target genes [12]. Loss or mutation in *NCOR2* gene is involved in chemoresistance and tumor growth [13]. However, to our knowledge, there have been no reports so far on the identification of *NCOR2-NTRK1* gene rearrangement in human cancers. According to the guidelines of The National Comprehensive Cancer Network (NCCN), there are several TKIs commercially available to target fusions of *NTRK1*, *NTRK2*, and *NTRK3* with varying degrees of activity [14]. Among these TKIs, larotrectinib, and entrectinib are the most common targeted agents tested in clinical trials and showed a significant impact on solid tumors [14]. NCCN guidelines and other studies have suggested that larotrectinib is a relatively potent and selective inhibitor against all three NTRK fusions [15]. Consistently, our case study confirmed that larotrctinib could effectively inhibit the tumor progression and development of this lung cancer patient with *NCOR2-NTRK1* fusion.

**Table 2:** Overview of the detected NTRK gene fusions in lung cancers

*ntrk* gene	Fusion partners	Tumor subtypes
*NTRK1*	*TPM3*	Adenocarcinoma[9]
	*MPRIP*	Lung cancer [10]
	*CD74*	Lung cancer [10]
	*SQSTM1*	NSCLC [11]
	*IRF2BP2*	NSCLC [12]
	*MPRIP*	Adenocarcinoma [13]
	*TPR*	Adenocarcinoma [13]
*NTRK2*	*SQSTM1*	Adenocarcinoma [9]
*NTRK3*	*ETV6*	Adenocarcinoma [9]
	*SQSTM1*	Squamous cell carcinoma [13]
		Neuroendocrine carcinoma [13]

*ERBB2* mutations were identified in this patient. *ERBB2* gene is a member of the epidermal growth factor (EGF) receptor family of receptor tyrosine kinases, which is commonly amplified and overexpressed in aggressive human cancers [16]. Currently, many tyrosine kinase inhibitors (TKIs) such as trastuzumab, pertuzumab, lapatinib, pyrotinib, and trastuzumab emtansine (T-DM1) have developed for targeting *ERBB2* amplification or overexpression in human tumors. However, the efficacy and sensitivity of these tyrosine kinase inhibitors for *ERBB2* mutations, excluding amplification and overexpression, are still waiting for further investigation. Indeed, although *ERBB2* mutation is a targetable driver in lung cancer, numerous studies reported the limited response rate and duration by using ERBB2 targeted therapies [17]. The potential resistance mechanisms may include *PI3KCA* mutation, *SRC* activation, *MET* overexpression, or other genomic alterations [17]. Due to the uncertainties of using ERBB2 targeted therapies, we decided not to use these inhibitors as the first targeted therapeutic option.

In this study, we used immunotherapy with PD-1 inhibitor of Camrelizumab and the targeted therapy with larotrectinib in this lung cancer patient. Regarding the treatment outcomes, larotrectinib had a marked response rate to target the tumor of this patient, indicating that novel *NCOR2-NTRK1* fusion plays a critical oncogenic driver in this tumor. However, targeted therapies using these inhibitors have limited duration of responses due to the acquired resistance formed eventually [18]. To solve this limitation, one of the effective approaches might be the combination of TRK and PD-1 inhibitors applied in the subsequent cancer treatment. Clinical evidence has indicated this type of therapeutic approach could significantly improve the long-term outcomes of *ALK* fusion-positive NSCLC [19]. Similarly, recent evidence has shown that NSCLC with *NTRK* fusion also has increased PD-L1 expression [11]. This raises the possibility of using the combined TRK and PD-1 inhibitors in this case with *NCOR2-NTRK1* fusion, positive PD-L1 expression, and high TMB simultaneously in the future.

## Data Availability

The data that support this case report are available from the corresponding author on reasonable request, since respecting the Ethics Committee to protect patient confidentiality.

## Abbreviations

TMB: Tumor mutational burden
PD-L1: Program death-ligand 1
IHC: Immunohistochemical
CT: Computed tomography
ICI: Immune checkpoint inhibitors
PD-1: Programmed death-1
NSCLC: Non-small cell lung cancer
NCCN: National Comprehensive Cancer Network
EGF: Epidermal growth factor
TKIs: Tyrosine kinase inhibitors
